# Overperceiving desire, underestimating age: a review of a narrow research lens

**DOI:** 10.3389/fpsyg.2026.1837065

**Published:** 2026-05-26

**Authors:** Anne Obrecht, Christian Agrillo

**Affiliations:** 1 Department of General Psychology, University of Padova, Padova, Italy; 2 Padua Neuroscience Center, Padova, Italy

**Keywords:** error management theory, interpersonal perception, participant sampling bias, sexual misperception, sexual overperception bias

## Abstract

The male sexual overperception bias describes the male tendency to sometimes misinterpret friendliness in women as sexual interest. Sexual overperception is a risk factor for sexual coercion and therefore deserves special attention in research, especially since differences in this bias across age groups may lead to differences in coercion risk across the lifespan. Previous research has mainly worked with undergraduate students as participants. Considering that other behavioral biases were found to change across the lifespan, this tendency in research was hypothesized to prejudice the results. We reviewed the literature in this field, revealing a mean participant age of 22.9 years across studies on the topic of sexual overperception, which points to the presence of a potential research bias as a function of participants’ age. As far as we are aware, no research efforts attempting to replicate previous findings in samples aged 40 + have been made. The implications of these findings are discussed as well as the possibility of sexual overperception bias as a universal phenomenon. We call for future investigation with older age groups as well as across different cultural contexts to form a broader picture in this field.

## 1 Introduction

The male sexual overperception bias describes the heterosexual male tendency to sometimes misinterpret friendliness in women as sexual interest. This bias was first established in 1982 (Abbey, 1982) and has since been an object of ongoing research. Numerous studies have established a link between sexual overperception and sexual predatory behavior and/or intent (e.g., Abbey et al., 1998, 2001; Farris et al., 2008a,b, 2010; Bouffard and Miller, 2014, 2023, 2024a,b; Neilson et al., 2025), which highlights the continued relevance of research into sexual overperception: It has direct implications for understanding and possibly preventing sexually aggressive behavior. Several theoretical perspectives have been proposed to explain this phenomenon.

According to an evolutionary perspective, the bias is supposed to be adaptive: Error management theory (EMT) suggests that overperceiving sexual interest would minimize missed mating opportunities, thereby increasing the male’s chances of reproductive success (Haselton and Buss, 2000). It was suggested that this bias is male-specific as the cost of missed mating opportunities was historically higher for men than for women (pregnancy, childbirth, infant care). Interpreting the phenomenon in a different rationale, cognitive explanations consider information-processing errors, proposing that attentional biases or limitations in cognitive skills may lead individuals to misread ambiguous social- or context clues, like smiling or clothing (Farris et al., 2008a; Treat et al., 2016). From a sociocultural standpoint, media portrayals, social scripts and gender norms may influence learned expectations about female behavior and sexual availability (Boyd-Rogers et al., 2022; Rodgers et al., 2023). It has also been proposed that the observed asymmetry between other-rated and self-reported sexual interest may, in part, reflect a tendency among women to underreport their own sexual interest (Perilloux and Kurzban, 2015; Livingston and Rerick, 2023). Furthermore, research on individual differences has identified several traits, such as rape-myth acceptance, traditional attitudes toward women, or high sexual drive as correlates of stronger overperception, particularly when combined with alcohol consumption (for a review see: Farris et al., 2008b; Bouffard and Miller, 2023).

Since its first discovery, a great deal of research has been conducted on sexual overperception bias. Many studies resulted in replications of the effect even across different cultures and research designs (e.g., Haselton, 2003; Bendixen, 2014; Kohl and Robertson, 2014; Hiraishi et al., 2016; Lee et al., 2019). However there has been some contrary evidence in specific samples; for example, men in long-term relationships actually tend to *under*perceive interest (Muise et al., 2016), and other research has failed to find a robust bias when controlling for individual differences in sexual openness (Lee et al., 2019) or self-reported past mating success (Yang and Zheng, 2022). This prompts the question: Can it be considered a universal phenomenon? The empirical basis to make such a claim is currently limited. Although the bias has been replicated both in Western and in non-Western samples (Bendixen, 2014; Hiraishi et al., 2016), recent evidence suggests that the phenomenon may not be as universal as previously thought: Within Western countries, cultural differences in the acceptance of female nudity may lead to variations in the presence of sexual overperception in specific situations (Widman et al., 2024). A comprehensive test of universality would require systematic investigation across diverse cultural, developmental, and situational contexts—an important goal for future research, but beyond the scope of this mini-review. The present work aims to focus on a specific aspect necessary for the evaluation of the universality of a phenomenon in human sexuality, namely the influence of age in the emergence of the sexual overperception bias.

## 2 Sexual overperception across the lifespan

### 2.1 Age-related shifts in behavior

Little is known about how sexual overperception manifests—or whether it persists—across the lifespan. This is notable, as other evolutionarily grounded phenomena, such as mate preferences or risk-taking behavior, have been shown to vary significantly with age and life stage: For example, younger men tend to prefer only slightly younger women in partner selection, while older men prefer increasingly younger women (Alterovitz and Mendelsohn, 2009; Gottfried et al., 2024). Women prefer somewhat older men when young and tend to prefer men closer to their own age as they get older (Alterovitz and Mendelsohn, 2009; Gottfried et al., 2024). Furthermore, risk-taking tends to decline or shift with age in many behavioral tasks (Mikels and Reed, 2009; Mata et al., 2011). Another example is the commitment skepticism bias—the tendency to underestimate other’s willingness to commit romantically—which was found in young and fertile women but not in postmenopausal women (Cyrus et al., 2011). Explanations for such changes have ranged from evolutionary principles to cognitive changes and societal influence. Given these age-related shifts, it is plausible that sexual overperception might vary similarly. Such variation could exist in magnitude, expression, or even in its presence.

### 2.2 Hypothesis

Within evolutionary psychology, EMT suggests sexual overperception persists as long as the reproductive benefits of a potential mating encounter outweigh the social or energetic costs of a false alarm (Haselton and Buss, 2000). While one might expect the bias to be most pronounced during peak fertility which declines with age, the specific prediction for older age is more complex. As male fertility declines, the relative “cost” of missed opportunities remains significant, but the adaptive value of pursuing scarce reproductive chances must be weighed against the increasing importance of resource conservation for existing kin (Hill and Kaplan, 1999). Therefore, while a linear decline in the bias across the lifespan is plausible, EMT could also predict persistence if the cost of a false alarm remains low (Haselton and Galperin, 2013). Thus, investigating older samples is critical to determine if this cost-asymmetry is a flexible response to a man’s current reproductive status or a stable, lifelong trait of male psychology. This distinguishes the evolutionary account from a cognitive and sociocultural perspective, which might attribute age-related declines mostly to accumulated social expertise or improved emotion-cognitive integration (Blanchard-Fields, 2007).

As Lindgren et al. (2008) stated, the majority of research published on misperceptions of sexual intent seems to draw on undergraduate students as study participants. Unfortunately, these results offer no indication as to whether they will hold up among other populations with an average of, say, 40 or 60 years of age: The influence of many extraneous variables seems plausible (see above).

The authors of this paper could not identify any research specifically aiming to replicate findings related to sexual overperception in older samples. A research bias towards samples of participants belonging to a rather narrow, specific age group was therefore hypothesized. To test this hypothesis, a quantitative synthesis of previously published research on the topic was conducted to establish the mean age of participants across studies.

## 3 Method

**Figure 1:**
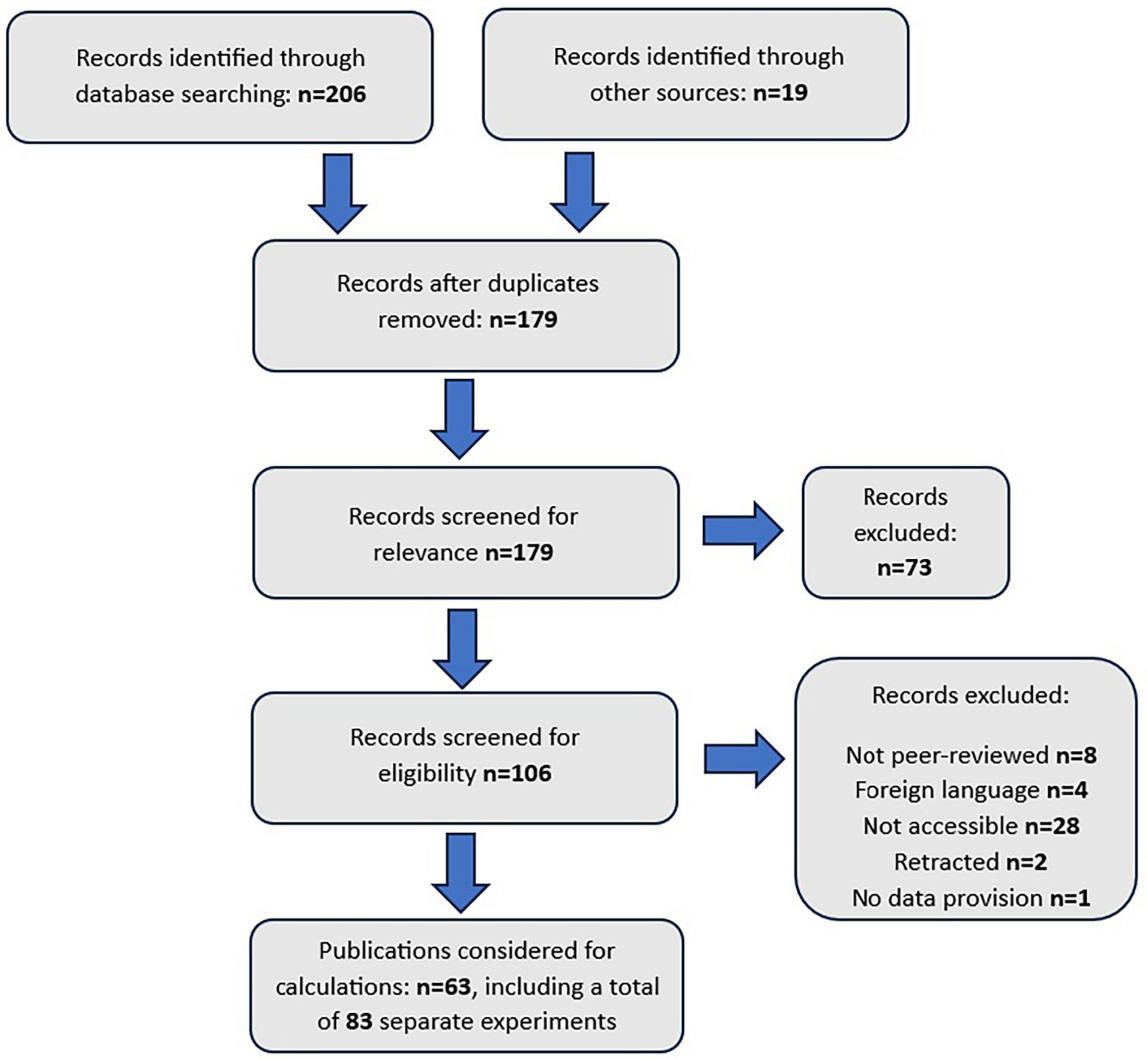
Flowchart of record selection following the PRISMA guidelines.

The PRISMA (Preferred Reporting Items for Systematic Reviews and Meta-Analyses) method was used to gather available data of relevant research (Figure 1). Searching separately with 21 different keywords and terms (Table 1), Google Scholar was searched. Given the impractical number of hits retrieved through broader queries, the Google Scholar search was limited to titles of publications. Random cross-checks were made with other databases, like PubMed and Semantic Scholar, to ensure that those did not entail further data. 206 records were identified through Google Scholar, 19 through other sources, like references found within publications. 46 duplicates were removed. Next, the identified records were screened for relevance to the topic of misperception of sexual intent, which led to 73 further records to be excluded. Only papers containing at least one empirical study were considered, meaning that literary reviews on the topic were discarded. In a next step, further records had to be eliminated: Publications that were not peer-reviewed in international journals (8), research published in languages other than English (4), records that only appeared as citations in Google Scholar but were not possible to come by online (28), one publication that only provided the participant age range and median, not allowing for a meaningful approximation of the average age, and two that had been retracted. Furthermore, three reports could not be accessed as they were not open access and we did not have a subscription for the journals, those therefore had to be excluded as well since the information in the abstract did not indicate which age demographic the participants belonged to. In 15 eligible publications, no numerical data was provided regarding participant age or the freely accessible abstract did not provide such data. However, in all of them it was specified that the participants were undergraduate students. Using this information, an approximation of the average age for the participants of those studies was calculated: The mean ages of participants from other eligible research stating that the participants were undergraduates were summed up and divided by the total number of such studies, leading to a rough estimation. This proxy (20.25), assumed to sufficiently accurately represent the actual (unknown) average participant age of the 15 mentioned publications, was assigned to those records as a substitute for the true mean participant age.

**Table 1:** List of keywords used to search Google Scholar for.

Search term
Sexual overperception bias
Sexual misperception
Sexual misperception bias
Overperceive sexual interest
Misperceive sexual interest
Perceptions of sexual intent
Misperception of sexual intent
Misperception of sexual interest
Misperceptions of sexual interest
Misperceive friendliness
Systematic bias sexual
Overperception of sexual intent
Overperception of sexual interest
Perceived sexual intent
Perceived sexual interest
Sexual overperception
Misperceive sexual intentions
Misperception sexual interests
Sexual or friendly
Sexual misperceptions
Overperceive sexual intent

As mentioned in 2.1.2, the search was limited to titles of records.

Finally, 63 publications were identified as eligible, containing in total 83 separate studies: Some papers included not just one, but up to four separate experiments. All of those were considered separately for the calculation since they were conducted on different samples. For an overview of records considered with their respective mean age see Table 2. The average age of participants was calculated by dividing the sum of age averages by the total of individual studies.

**Table 2:** Overview of records considered for the quantitative synthesis, alphabetically sorted by author name.

Authors, year	Study focus	Participant age		
		Range	Mean	SD
Abbey (1982)	Establishing the bias		20.25	
Abbey and Melby (1986)	Nonverbal cues		20.25	
Abbey (1987)	Clothing, sex composition		20.25	
Abbey and Harnish (1995)	Rape supportive attitudes, alcohol	18–21	20.25	
Abbey et al. (1998)	Sexual assault, alcohol	18–59	20.25	
Abbey et al. (2000)	Alcohol		25.7	5.6
Abbey et al. (2001)	Sexual Assault	18–53	20.25	
Abbey et al. (2003)	Alcohol consumption		26.4	7.8
Abbey et al. (2005)	Alcohol, past sexual assault		25.6	3.6
Bendixen (2014)	Norwegian culture		22.6	
Bibby et al. (2023)	Accurately detecting facial expressions		19.7	
Bondurant and Donat (1999)	Affective attitudes, acquaintance rape		20.00	
			19.90	
Bouffard and Miller (2014)	Sexual coercion		22.00	
Bouffard and Miller (2023)	Rape myth acceptance, arousal		21.70	
Bouffard and Miller (2024a)	Sexual coercion intent		20.25	
Bouffard and Miller (2024b)	Misperceptions and sexual coercion in women		20.25	
Boyd-Rogers et al. (2022)	Social cognitive processes		19.00	1.45
Burch and Widman (2024)	Nipple erection		19.24	
Brandner et al. (2021)	Signal detection theory	18–26	19.05	1.41
		18–82	44.36	13.84
Cahoon and Edmonds (1989)	Women’s clothing style		20.25	
Chen et al. (2025)	Sexual interest perception		24.50	
De Jong and Reis (2015)	Variables in same-sex couples	18–75	28.08	9.81
DeSouza et al. (1992)	Brazilian culture		20.25	
Bowman Edmondson and Conger (1995)	Different media	18–23	20.25	
			20.25	
Edwards and Vogel (2015)	Exposure to rape conducive-norms		20.10	
Farris et al. (2008a,b)	Perceptual sensitivity vs. decisional bias		19.60	1.72
Farris et al. (2010)	Affective cues, clothing cues		19.60	
Fichten et al. (1992)	Verbal and non-verbal cues	18–45	26.00	
Fisher and Walters (2003)	Various variables	18–43	19.60	
Goetz et al. (2024)	Exogenous testosterone exposure		23.00	5.91
Hall et al. (2015)	Flirting	18–30	19.20	2.1
		18–47	19.60	3.29
Harnish et al. (1990)	Self-monitoring		20.25	
Haselton and Buss (2000)	Siblings, various		18.59	
			19.18	
Haselton (2003)	mate value		19.17	
Henningsen (2004)	Flirting motivations		20.25	
Henningsen and Henningsen (2010)	Own sexual interest		19.96	
Hiraishi et al. (2016)	Japanese culture		21.98	1.85
Howard and Perilloux (2017)	Homosexuality	18–74	32.95	
Howell et al. (2012)	Sociosexuality		23.00	3.6
Hynie et al. (2003)	Condom posession		24.22	6.86
Jacques-Tiura et al. (2007)	Attitudes, past experiences	19–48	24.00	
Koenig et al. (2007)	Friendships		18.79	1.25
			18.85	0.71
Kohl and Robertson (2014)	Mate value		24.24	
Kowalski (1993)	Behavioral cues		20.25	
			20.25	
Kunstman and Maner (2011)	Power		18.70	1.18
			19.00	
			19.00	
			19.20	
Lee et al. (2019)	Own level of sexual interest		19.77	2.88
Lenton and Bryan (2005)	Sexual scripts	18–22	18.79	
		18–26	18.98	
Lindgren et al. (2007a)	Implicit associations test		19.09	1.31
			20.26	3.13
Lindgren et al. (2007b)	Perceiver experience		19.02	0.9
			19.11	1.04
Livingston and Rerick (2023)	Women’s ratings of sexual intent		42.46	12.89
Moran and Burch (2023)	Diverse sexual orientations	20–74	34.27	10.82
		23–69	36.96	12.8
		18–64	27.86	9.37
Muise et al. (2016)	Long-term relationships	23–61	36.00	8.7
		18–68	23.64	8.21
		18–53	26.00	7
Neilson et al. (2025)	Alcohol intoxication		24.71	
Perilloux et al. (2012)	Sociosexuality, mate value		18.70	1
Perilloux and Kurzban (2015)	Women’s true sexual interest	18–75	31.80	11.7
		18–82	31.09	10.92
		18–75	32.41	11.67
Samara et al. (2021)	Arousal, own sexual interest		22.31	
Shea (1993)	Sexual coercion	18–40	22.00	4.06
Sigal et al. (1988)	Romantic priming		20.25	
			20.25	
Treat et al. (2016)	Environmental context	18–24	19.32	1.35
Wagstaff and Van doorn, (2018)	Schizotypy and clothing color	18–63	31.13	11.08
Wegner (2014)	Alcohol, testosterone		24.30	
Widman et al. (2024)	Nipple erection, cultural comparison		22.10	1.92
Yang and Zheng (2022)	Mating confidence, mating efficacy		26.40	
		AVG:	22.87	

Range and standard deviation are listed only if they were provided in the cited study. The cells in the “mean” column in dark grey contain an approximation (20.25), since no mean age was indicated in the study, only the information of participants being undergraduate students (see 3). Some records contained several separate experiments, the data for each additional study are listed in a separate row below the respective paper. All above mentioned research can be found in the references. The light grey shading serves to separate publications for better readability.

## 4 Results

**Figure 2:**
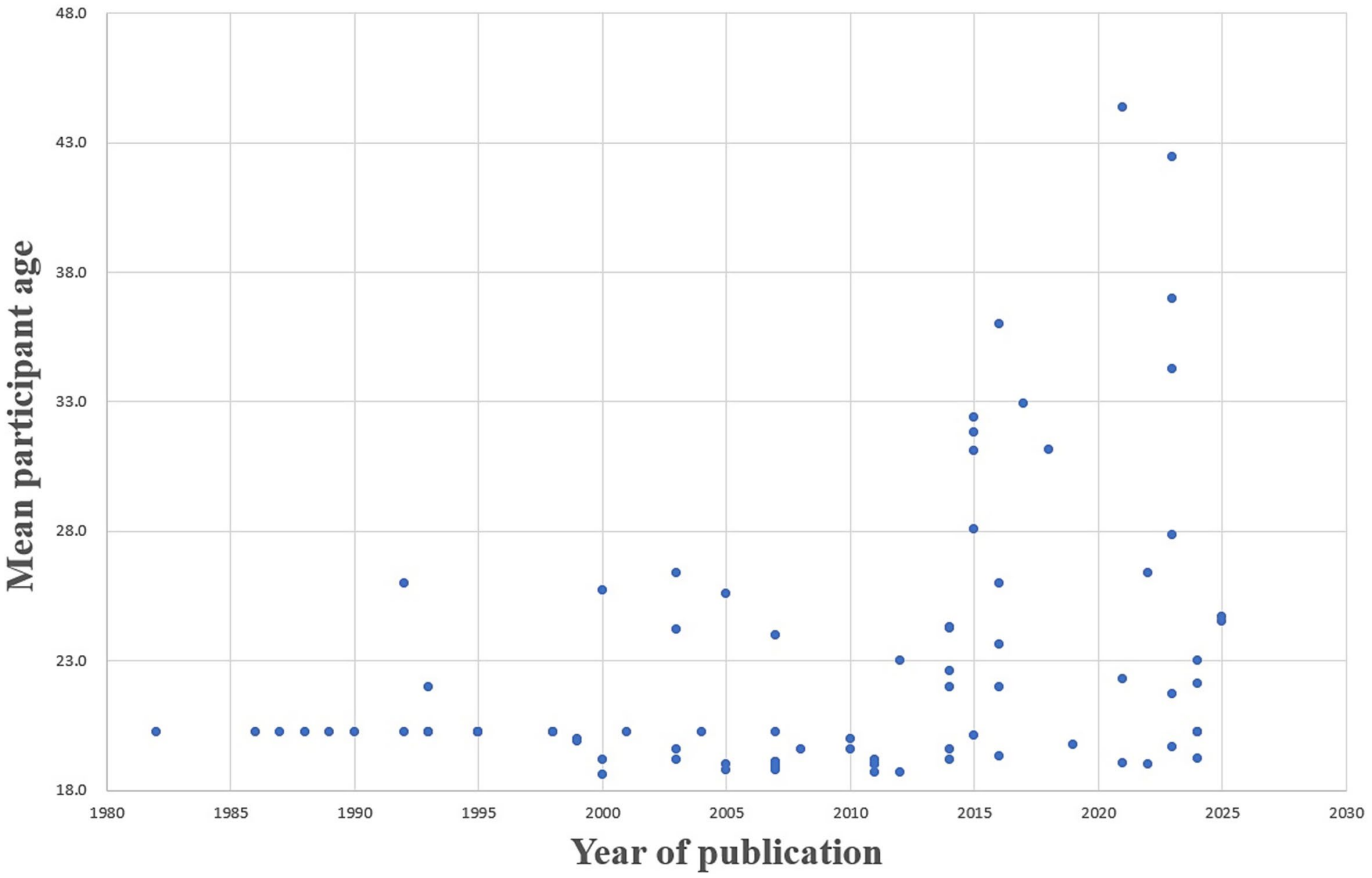
Scatter chart of participant mean age and research publication year.

A mean age of 22.87 across all eligible studies was found [Median = 20.25, Range = (18.59, 44.36), SD = 5.4]. This observed standard deviation indicates a considerably narrow distribution, with approximately 68% of the samples considered falling within a tight 10.8-year window between the ages of 17.45 and 28.29. This concentration confirms that the empirical foundation of the field heavily relies on young adult populations. To test for the impact of using an approximated age (20.25) for 15 studies, a sensitivity analysis was conducted. This resulted only in a slight increase both in mean age (23.6) and standard deviation (5.9) compared to the full sample, which suggests that the inclusion of approximated data did not significantly distort the overall age distribution of the synthesis. Before 2014, studies were exclusively conducted with participants that averaged in age between 18 and 26 years. Since then, several studies have instead opted for participants averaging between 30 and 40 years, with one study even reaching a mean age of 44.36 years (Brandner et al., 2021). To assess chronological shifts in demographics, a linear regression was conducted with publication year as the predictor and mean participant age as the outcome (see Figure 2). The model was significant [*F*(1,81) = 13.49, *p* < 0.001, *R^2^* = 0.14], indicating a positive correlation (*r* = 0.38) between publication year and participant age. The regression coefficient (*β* = 0.18) indicates that while there is an increase in participant over time, such change is slow. In fact, 68% of studies conducted from 2014 on continue to test participants with an average age of 26.4 or lower (out of those: range = [19.0, 26.4], average age = 21.8).

## 5 Discussion

### 5.1 Variables that may determine changes across the lifespan

In support of the hypothesis, the average age of 22.87 points to a research bias in sexual misperception research, with the bulk of research being conducted primarily on samples of undergraduates. This narrow age range (17–28) represents only 13.5% of the U.S. population (U.S. Census Bureau, 2023), where most studies were conducted, creating a significant “representativeness gap.” While our trend analysis confirms a recent, statistically significant tendency towards using older samples (*p* < 0.001), this is a modest shift, and deliberate recruitment of older participants is necessary. Specifically, replication attempts of previous findings should be made with participants aged 40 + to test competing theoretical accounts for the phenomenon. From an evolutionary perspective, EMT predictions for older age remain open to debate. One possibility is a down-regulation as male reproductive value declines (see 2.1.1). However, if the cost of a “false alarm” remains low, the bias might persist or even increase to make sure the few reproductive opportunities are not missed (Haselton and Galperin, 2013). In contrast, Socioemotional Selectivity Theory (SST) predicts that motivational goals may change across the lifespan (Carstensen et al., 1999). It posits that as people age and perceive their remaining time as more limited, their goals shift to prioritize emotional intimacy and relational stability over novelty-seeking and exploraty behavior. This could lead to lower overperception in new encounters but higher accuracy in established ones, representing a qualitative shift in social goals rather than a simple change in bias magnitude. Finally, accumulated life experience, particularly of the interpersonal type, might moderate the extent of sexual overperception in older samples. Experiential influences have been theorized in other age-related behavioral changes, for instance improved emotion regulation later in life, which has partly been attributed to the coping strategies and emotional knowledge acquired over the lifespan (Yeung et al., 2011). Older, more experienced participants might therefore detect sexual interest more accurately (Isidori et al., 2005; Wu et al., 2022; Catena et al., 2024; Carstensen et al., 1999). All in all, there are several accounts that predict different trajectories of sexual misperceptions across the lifespan, and this developmental context should be considered in future research.

### 5.2 Findings of research with older participants

More recently, some researchers made efforts to include study populations besides the usually employed range of 18–25 years of age (e.g., Perilloux and Kurzban, 2015; Livingston and Rerick, 2023; Moran et al., 2023). While the aim of most of these studies was not to specifically replicate previous findings in different demographic groups, with a participant mean age ranging from 30 to 45, the results of this research have more external validity, meaning generalizability to the general population, than those including only college students. This is because with a range of 18–70 and the beforementioned average, they come rather close to the average US population (where many of these studies are conducted), at least in terms of age: In 2022, 64.91% of US citizens were between 15 and 64 years old (O’Neill, 2024), with a median age of 38.9 years old (Vasquez, 2023). While these statistics must not be directly compared to the mean age the participants had, they give a rough idea of the average age distribution in the U. S., which approaches that of the participants.

Which results did research with older samples bring? Perilloux and Kurzban (2015) were able to replicate Haselton and Buss’ (2000) findings of the typical sex difference in ratings of sexual intent in a sample with an average age of 31.8 years (SD = 11.7; Range = 18–75). Although this knowledge is valuable, replication attempts in significantly older samples than this one have not been published to date. Other research with older participants was focused on investigating specific variables in relation to sexual overperception, not on replications. In an interesting attempt to establish a new kind of measurement for the sexual overperception bias, Brandner et al. (2021) conducted two studies with a similar design but different age groups (average = 19.05 in the first and 44.36 in the second study). Unfortunately, the stimulus materials were changed simultaneously with the participant demographic in the second experiment. The researchers found an increase in sexual misperception in the older group. However, since the stimuli were adapted to be more ambiguous for this group, such differences in perception cannot safely be attributed to the age difference, as they might simply be a result of increased stimulus ambiguity. A study by Livingston and Rerick (2023), conducted on women (mean age = 42), found evidence for an actor-observer asymmetry in judgments of sexual interest: Female participants rated other women’s interest in a male target as higher than their own, particularly when the target was attractive. This asymmetry may partly explain misperceptions of women’s sexual intent by observers. In a set of both quantitative and qualitative research, sexual misperceptions were investigated among LGBTQ samples (De Jong and Reis, 2015; Howard and Perilloux, 2017; Moran et al., 2023), with participants averaging 33, 28 and 30 years in age. The results led to the conclusion that non-heterosexual mating strategies are more complex than a simple continuation of heterosexual mating strategies or an inversion of gender roles. Further research has found that men might actually tend to under-, not overperceive sexual interest in long-term relationships (Muise et al., 2016) (mean age = 36), and that high-schizotypy individuals overperceive sexual interest when seeing women wearing red (Wagstaff and Van doorn, 2018) (mean age = 31.13). It is good to see this more recent tendency to conduct research on samples outside of one specific age group and cultural context. However, while these are all important and meaningful findings, research aiming to specifically investigate the existence or magnitude of sexual overperception among older samples (40 + years) is still lacking.

### 5.3 Future research

Specifically, it should be interesting to investigate sexual overperception in older men (Harris et al., 2011). As detailed in 3.1, different explanations of sexual overperception come with different predictions for such experiments. Research with older participants could therefore advance our knowledge of the phenomenon and provide support or disconfirmation for specific accounts.

Future research with older participants must ensure ecological validity of the stimuli used. Methodologies in current overperception research differ: A research design where a dyad (male–female) interacts and subsequently rates own and other-perceived sexual interest seems applicable in any age group, as well as settings where third raters observe such interactions and rate perceived sexual interest of both participants. However, research designs using vignettes (be that imaginary, written, photos or videos) must include carefully adapted stimuli that resonate with middle-aged or older men, as current vignettes may be more matched to fit younger participants due to the particular settings, protagonist characteristics and interactions they use. Future studies should therefore first validate such new stimuli among the target age group to avoid confounding results by measuring age-incongruent targets.

Most importantly, the link between sexual misperception and sexual violence has been established repeatedly (Abbey et al., 1998; Farris et al., 2008b; Bouffard and Miller, 2023, 2024a; Neilson et al., 2025). While sexual coercion is a serious issue among college students, it is not limited to this population (Mumford et al., 2020). U.S. adults who never attended college report harassment and assault at rates similar to those who did (e.g., ~32.7% harassment, ~24.6% assault) (Mumford et al., 2020), and up to 3.1% of older adults (60 + years) in Europe reported having experienced sexual violence in 2019 (Nobels et al., 2020). Because sexual coercion is not confined to early adulthood, the “representativeness gap” identified in this review presents a problem with practical and societal implications: Since sexual overperception seems to drive sexual violence, and sexual violence occurs across the lifespan, we need to investigate and understand sexual overperception across the lifespan. Further research with different age groups is therefore crucial. We propose that the most impactful future direction would be a longitudinal or cross-sectional study explicitly testing whether sexual overperception in older men remains a reliable predictor of coercive attitudes or behaviors. Such research would ideally include contextualized vignettes and potential moderators that are age-relevant, such as relationship status, or cognitive factors derived from SST. Furthermore, distinguishing between magnitude of bias and behavioral intent would determine if older men possess a similar cognitive bias as younger men do, but express it differently due to higher social cost or accumulated life experience.

By understanding if the cognitive mechanisms that drive sexual aggression are universal across the lifespan or not, age-specific intervention strategies can be developed.

## 6 Conclusion

Returning to the original question: Is the sexual overperception bias a universal phenomenon? At least regarding development across the lifespan, further evidence is required. Importantly, universality does not imply that a phenomenon manifests identically across all populations (Norenzayan and Heine, 2005): A bias can be universally present while still being shaped in its expression by factors such as culture, life experience, or developmental context.

As Lindgren et al. (2008) note, most sexual misperception research has focused not only on college students as participants, but they also tend to be white and from the US. However, WEIRD (Western, Educated, Industrialized, Rich, and Democratic) societies are among the least representative globally (Henrich et al., 2010). To assess the true scope and mechanisms of the bias, future studies should examine whether and how the bias varies across social, cultural, developmental, and cognitive contexts. A more nuanced understanding of when and for whom this bias occurs could help inform targeted interventions to reduce the risk of misperception escalating into sexual violence.

### 6.1 Limitations

Restricting the search to titles, we may have excluded studies that used different terminology or measured sexual overperception as a secondary variable. However, we are confident that the 63 included publications represent the core literature in this field. While it is possible that broader social-cognitive studies, which may include more diverse age groups, were missed, the consistent trend toward undergraduate samples in the retrieved data suggests it is unlikely that any missing research would significantly shift the mean age away from the identified young adult demographic. Three relevant studies could not be considered due to accessibility issues, though it seems unlikely that they could have considerably impacted the overall mean. Some age approximations were required due to missing data, which may introduce minor inaccuracies. However, sensitivity analyses revealed that these approximations did not significantly affect the mean or the variance of the sample.
